# Delivery preferences for psychological intervention in cardiac rehabilitation: a pilot discrete choice experiment

**DOI:** 10.1136/openhrt-2021-001747

**Published:** 2021-08-23

**Authors:** Gemma Elizabeth Shields, Stuart Wright, Adrian Wells, Patrick Doherty, Lora Capobianco, Linda Mary Davies

**Affiliations:** 1 Manchester Centre for Health Economics, The University of Manchester, Manchester, UK; 2 School of Psychological Sciences, The University of Manchester, Manchester, UK; 3 Research and Innovation, Greater Manchester Mental Health NHS Foundation Trust, Manchester, UK; 4 Department of Health Sciences, University of York, York, North Yorkshire, UK

**Keywords:** cardiac rehabilitation, health care economics and organizations, delivery of health care, telemedicine, research design

## Abstract

**Background:**

Cardiac rehabilitation (CR) is a programme of care offered to people who recently experienced a cardiac event. There is a growing focus on home-based formats of CR and a lack of evidence on preferences for psychological care in CR. This pilot study aimed to investigate preferences for delivery attributes of a psychological therapy intervention in CR patients with symptoms of anxiety and/or depression.

**Methods:**

A discrete choice experiment (DCE) was conducted and recruited participants from a feasibility trial. Participants were asked to choose between two hypothetical interventions, described using five attributes; intervention type (home or centre-based), information provided, therapy manual format, cost to the National Health Service (NHS) and waiting time. A separate opt-out was included. A conditional logit using maximum likelihood estimation was used to analyse preferences. The NHS cost was used to estimate willingness to pay for aspects of the intervention delivery.

**Results:**

35 responses were received (39% response rate). Results indicated that participants would prefer to receive any form of therapy compared with no therapy. Statistically significant results were limited, but included participants being keen to avoid not receiving information prior to therapy (β=−0.270; p=0.03) and preferring a lower cost to the NHS (β=−0.001; p=0.00). No significant results were identified for the type of psychological intervention, format of therapy/exercises and programme start time. Coefficients indicated preferences were stronger for home-based therapy compared with centre-based, but this was not significant.

**Conclusions:**

The pilot study demonstrates the feasibility of a DCE in this group, it identifies potential attributes and levels, and estimates the sample sizes needed for a full study. Preliminary evidence indicated that sampled participants tended to prefer home-based psychological therapy in CR and wanted to receive information before initiating therapy. Results are limited due to the pilot design and further research is needed.

**Key questions**

**What is already known about this subject?**

- The current evidence base of preference research in cardiac rehabilitation (CR) is heterogeneous in design and study sample and focuses on educational and exercise sessions. It indicates that preferences for home-based CR differ across participant groups.

**What does this study add?**

- In a sample of people who had symptoms of anxiety and depression following a cardiac event, the study adds evidence on preferences for the delivery of psychological therapy as part of CR and demonstrates that research of this type is feasible. Findings suggest that participants may prefer home-based options, and would like information given prior to starting therapy, but further research is needed.

**How might this impact on clinical practice?**

- The research indicated that participants were keen to receive psychological therapy as part of CR (based on response to opt-out). We recommend that practitioners are mindful of patient preferences when offering psychological therapy to CR patients.

## Introduction

The burden associated with cardiovascular diseases globally is considerable; it is the leading cause of mortality and a key cause of disability.^1^ The burden is increasing, with prevalent cases of cardiovascular disease doubling between 1990 (271 million cases) and 2019 (523 million).^1^ Subsequently, there is a need for effective and cost-effectiveness healthcare interventions for affected populations. Cardiac rehabilitation (CR) is a supervised programme offered to people following a cardiac event and comprising of exercises, education and psychological care.^2^ Evidence suggests that CR reduces morbidity and improves quality of life, and is often cost-effective.^2 3^

In the UK, around 90 000 people start CR annually (2019 figures).^4^ Three-quarters of CR participants in the UK access group-based supervised CR at a healthcare centre.^4^ Though there is evidence to suggest centre-based and home-based delivery modes have equivalent outcomes, a minority of participants have home-based CR (8.8%).^4 5^ On entry to CR around 30% of people have symptoms of anxiety and around 20% have symptoms of depression.^4^ Therefore, CR programmes are uniquely placed to provide vital psychological interventions. A meta-analysis identified that psychological interventions added to exercise-based CR were associated with a reduction in symptoms of depressions and cardiac morbidity.^6^ As centre-based and home-based CR are available, psychological intervention is needed across delivery modes.

Discrete choice experiments (DCE) are increasingly used to elicit preferences for healthcare interventions and services.^7 8^ Within a DCE, participants make choices between hypothetical scenarios that are summarised using key attributes. Each attribute has several levels that account for how the attribute can vary. Stated preference methods are based on the assumption that healthcare interventions and services can be described by a number of attributes and that an individual’s valuation of that intervention/service will vary according to their preferences for levels of those attributes.^9^ Responses allow researchers to quantifiably elicit stated preferences.

The current evidence base for preferences in CR is methodologically heterogeneous and focuses on exercise and education activities. Boyde *et al*, found preferences were strongest for centre-based programmes providing timely group exercise sessions and one-to-one educational sessions.^10^ While the results indicated that technology delivered exercise and educational sessions would be less popular, preference heterogeneity was noted, and authors discussed that a one-size-fits-all approach may be unsuitable. Kjaer and Gyrd-Hansen reported preference heterogeneity when they focused on preferences for CR activities.^11 12^ Activities included physical exercises, personal meetings with a nurse, group counselling, diet guidance and smoking cessation. The authors found that personal meetings were preferred, followed by physical exercise, and nutritional counselling. Preferences differed by gender, and older people (especially men) did not value the offer of CR highly. Whitty *et al*, found home-based chronic heart failure management plans were preferred by older people, those with a lower income and people living alone.^13^ Tang *et al*, found people who preferred a home-based setting for CR reported better physical health and exercise capacity.^14^ Overall, the existing evidence suggests preferences for home-based CR is heterogeneous.

There has been a significant increase in home-based CR mode of delivery (23%–59%) due to COVID-19 service adaptation.^15^ Given the growing focus on home-based CR formats and the lack of evidence on preferences for psychological care in CR, there is a clear need to investigate patients’ preferences for the design of home-based CR to inform future research and intervention design. The present study is part of an National Institute for Health Research-funded programme called PATHWAY aimed at improving psychological outcomes in CR patients using group-based or home-based metacognitive therapy.^16–18^

The PATHWAY Home-MCT feasibility single-blind randomised controlled trial (RCT) investigated the acceptability of delivering metacognitive therapy (MCT) in a home-based format for CR participants with symptoms of anxiety and depression.^16^ The trial recruited people referred to UK National Health Service (NHS) CR programmes with a score of ≥8 in the anxiety and/or depression subscales of the Hospital Anxiety and Depression Scale. Further details on the design and delivery of Home-MCT are available in the trial protocol.^16^ Results of the trial, which will be published separately, will provide evidence on the acceptability and feasibility of delivering the home-based format for metacognitive therapy for a sample of CR participants. The current study is an extension of the feasibility study; a pilot discrete choice experiment recruited from trial participants to investigate preferences for the delivery of psychological therapy in CR.

### Aims and objectives

The primary aim of this study was to explore the preferences of participants in the Home-MCT feasibility study, who have experienced a cardiac event and have symptoms of anxiety and/or depression, for attributes of a psychological therapy intervention in patients. Specific objectives were to:

- Identify which attributes were most important to patients.
- Evaluate the feasibility of recruiting from a trial sample.
- Estimate the sample size needed for a full study.

The results of this pilot DCE will help to inform the design of future studies to explore the preferences of patients for psychological interventions.

## Methods

The study used a DCE to examine preferences for psychological therapy in CR.

### Attributes and levels

The DCE design (attributes and levels) was constructed following a review of qualitative feedback from the wider programme of work, discussion with the Patient and Public Involvement (PPI) group and through iterative discussions with the trial research team (including clinical experts).^19–21^ Each hypothetical scenario included five attributes: (1) psychological intervention type (focusing on home-based or centre-based), (2) information provided prior to treatment, (3) therapy manual format, (4) cost to the NHS and (5) waiting time. The full characteristics and levels included in the discrete choice experiment design are included in table 1.

**Table 1:** Characteristics and levels

Attribute	Levels
1. Psychological intervention to be received alongside your standard cardiac rehabilitation programme.	Home-based psychological therapy using a manual with occasional telephone support from a healthcare professional. Home-based psychological therapy using a manual with occasional face-to-face support from a healthcare professional. Group psychological therapy based in primary or community care (eg, a local general practitioner or NHS clinic) delivered by a healthcare professional. Group psychological therapy based in secondary care (eg, at a hospital) delivered by a healthcare professional.
2. The information given to you prior to accepting and starting treatment that gives you an idea of what to expect from the therapy.	No information provided. A printed leaflet of information. An overview of the therapy from a healthcare provider with a chance to ask questions. An overview of the therapy from a healthcare provider with a chance to ask questions and a printed leaflet.
3. Format of the therapy manual and exercises.	Printed (paper copy) of the therapy manual and an accompanying audio CD of exercises. Printed (paper copy) of the therapy manual and an accompanying DVD (video) of exercises. Printed (paper copy) plus a website based manual and exercises. Printed (paper copy) plus a smartphone application-based manual and exercises.
4. Additional cost to the NHS.	£0. £500. £1000. £2000.
5. Programme start.	Within 2 weeks of hospital discharge. Within 4 weeks of hospital discharge. Within 6 weeks of hospital discharge. Within 8 weeks of hospital discharge.

NHS, National Health Service.

The first qualitative attribute was chosen to investigate preferences around whether participants would like to receive home-based or centre-based therapy. The second and third attributes were included as it was judged to be important by the PPI group and because this is something that could easily be addressed in practice if participants did prefer a certain level. Two quantitative attributes were included. Additional cost to the NHS was used to estimate willingness to pay for aspects of design/delivery of psychological therapy in CR, recognising that costs can be a key driver in healthcare decision-making. Programme start captured waiting times, which has been noted to be important in the literature in relation to enrolment/attendance and health outcomes.^22–24^

### Experimental design

The number of attributes (five), combined with the number of levels (four), gives a large number of scenarios to describe all the possible different combinations of the features of the therapy (estimated as the number of levels to the power of the number of characteristics). For example, a stated preference survey that uses five characteristics with four levels per attribute gives a total of 1024 possible profiles and over 500 000 potential DCE questions. It is not feasible to design a survey to assess preferences for all of these scenarios.

To address this, a fractional factorial design was used, to reduce the number of scenarios by selecting a sample of possible combinations that covers the combinations and effects of interest.^25^ A published design catalogue (http://neilsloane.com/oadir/oa.16.5.4.2.txt) and modulo arithmetic were used to generate an efficient, orthogonal fractional factorial design.^26^

**Figure 1:**
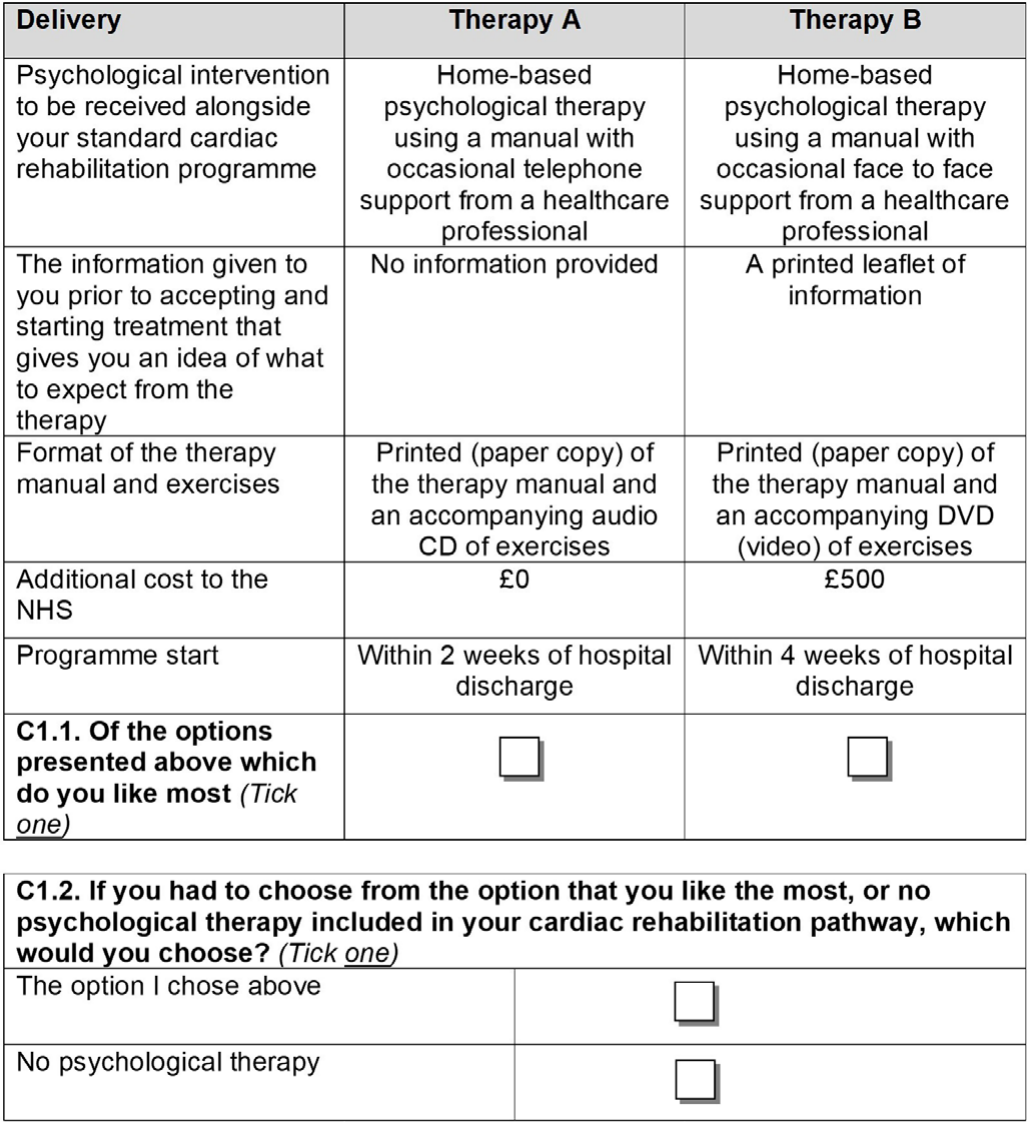
Example question. NHS, National Health Service.

A further question asked participants to choose between their preferred scenario and no psychological therapy (opt-out). Opt-out options are helpful in discrete choice experiments because they are more reflective of real life and can help with the interpretation of results.^25^ An example question is provided in figure 1.

Each survey consisted of four sections; basic socio-demographic details (age, gender, ethnicity, employment status and education level), the EQ-5D-5L, the DCE questions (known as choice sets) and a final section for the participants to provide additional information on how long the survey took and to collect any feedback. Participants were not obligated to answer all questions and could choose to skip questions, or in the case of demographics select ‘prefer not to answer’. A copy of the survey is included in the online supplemental material 1. EQ-5D values (utility values) were estimated using the crosswalk mapping algorithm, in line with current guidance from the National Institute for Health and Care Excellence.^27 28^

10.1136/openhrt-2021-001747.supp1 Supplementary data

### Recruitment

All participants in the Home-MCT feasibility trial, who had provided informed consent to take part in the trial, and consented to be invited to further research opportunities, were invited. Full trial inclusion and exclusion criteria are reported in the trial protocol.^16^ In brief, participants had to be adults eligible for CR and have clinically significant symptoms of anxiety and/or depression, with a competent level of English language skills.^16^ Participants were sent a paper copy of the survey materials (invitation letter, participant information sheet, instructions, consent to further contact form and questionnaire) by post (with a prepaid return envelope). The invitation letter also contained an online link (set-up using Select Survey) and participants could choose to complete a paper version or an online version. The stated preference survey was sent to participants after their final trial follow-up had been completed. Each participant was offered a £5 high street gift voucher to acknowledge participation in the DCE study.

### Sample size

Calculating a sample size for a DCE is challenging and researchers need to be confident that the sample size is large enough to detect differences in preferences.^29^ A range of factors need to be considered, including the DCE design (eg, number of attributes and choice sets), as well as preference heterogeneity.^29^ The sample in this pilot survey was restricted to participants in the Home-MCT trial. The results from this pilot study were used to estimate the sample size that would be required to calculate significant preference coefficients in a full DCE study. The estimated minimum sample size required was generated for D-efficient and Bayesian designs in the experimental design software NGene.^30^

### Statistical analysis

The data was analysed using individual choice responses as the dependent variable in the model.^31^ A conditional logit using maximum likelihood estimation was used in the first instance. The coefficients for each attribute indicate the direction of preference for that attribute. Qualitative attributes were effects coded.^32^ The continuous variables were included as linear in the baseline analysis. Tests for non-linearity of these variables were conducted by effects coding the variables and conducting Bayesian Information Criterion (BIC) tests on the non-linear and linear models.^33^ A BIC test was also conducted to test whether one constant could be used to represent preferences for therapy compared with opting out or whether separate constants for Therapy A or B were more appropriate.

The marginal rates of substitution (MRS) for each characteristic were calculated to estimate the relative utility of the characteristics. The MRS for each characteristic was estimated by dividing the coefficient for that characteristic by the inverse of the coefficient for the NHS cost characteristic. This allowed the estimation of the relative cost participants were willing to accept for the different features and assess how important they are (ie, Willingness to Pay). The results for each characteristic of the delivery of psychological therapy indicate how important users felt it was in CR. These data will be used to help inform future policy and commissioning decisions. Due to the small sample size in this pilot study, heterogeneity in preferences for the therapy design was not explored in this analysis.

## Results

### Response rate

Eighty-nine participants from the Home-MCT trial were invited to participate. The first invitation was sent on 5 March 2020, and 20 responses were received. Given the the COVID-19 pandemic at this time, a reminder was sent to participants who had not completed the survey on 30 June 2020 and subsequently a further 15 responses were received. In total, of the 89 participants invited, 35 responses were received (39% response rate). The online survey submission option was unpopular, with only one response received via this means.

In total 80% of participants (28/35) responded to all choice sets and 82.9% (29/35) responded to all opt-out questions. In total, over three-quarters (27/35, 77.1%) of participants had complete data.

### Participant demographics

An overview of DCE participant characteristics is reported in table 2. Note that for the pilot trial the mean age of participants was 59 (SD 7; range 40–84) and 36% were women.

**Table 2:** Participant demographics

	n	%
Gender		
Female	13	37.1
Male	22	62.9
Age, years		
45–54	4	11.4
55–64	14	40.0
65–74	16	45.7
75–84	1	2.9
Ethnicity		
White	34	97.1
Asian/Asian British	1	2.9
Education		
GCSE (or equivalent) or above	26	74.3
No GCSE (or equivalent)	7	20.0
Missing	2	5.7
Employment status		
Paid employment (including part-time or full-time)	11	31.4
Unpaid employment/activities (including voluntary employment, study, housewife/husband, retired)	22	62.9
Unemployed	2	5.7
Prior experience of psychological therapy		
Previously received psychological therapy (group or one-to-one)	6	17.1

GCSE, General Certificate of Secondary Education.

Participants had been experiencing cardiac problems for a mean of 5.2 years when they completed the questionnaire, and the most reported cardiac problem was a myocardial infarction (heart attack) which was reported by 22 participants (62.9%). The number of participants who reported having family affected by cardiac problems (15, 42.9%) was equal to the number reporting the opposite.

The mean EQ-5D value, which represents health status, was 0.64 (SD 0.27). This is lower than the population norms for people of a similar age; which are 0.804 for the group aged 55–64 and 0.785 for the group aged 65–74.^34^ A breakdown is reported in the online supplemental material 1.

### Participant preferences for a psychological intervention

Table 3 reports the results of the conditional logistic regression analysis. The BIC tests suggested that non-linear coding of the continuous variables did not add sufficient explanatory power and so linear terms were used. The test also suggested that a model with a constant term for each therapy rather than a single opt in constant was superior. As the constant for Therapy A is larger than that of Therapy B, there is potentially some evidence (ie, a larger constant) that participants were disproportionately likely to select the left-hand option, although this was not statistically significant.

**Table 3:** Conditional logistic regression results

Attribute/level	Coefficient	P value	Willingness To Pay £ (95% CIs)
Psychological intervention to be received			
Home-based with telephone support	0.076	0.528	98 (−208 to 404)
Home-based with face-to-face support	0.102	0.405	131 (−179 to 441)
Group face-to-face in primary or community care	−0.085	0.493	−109(−422 to 204)
Group face-to-face in secondary care	−0.093	0.444	−120(−428 to 189)
Information given prior to starting treatment			
No information	−0.270*	0.033	−348(−674 to −21)
Printed leaflet	−0.001	0.992	−2(−299 to 296)
Overview from a healthcare professional	0.103	0.394	133 (−174 to 440)
Overview from a healthcare professional plus printed leaflet	−0.165	0.184	−213(−530 to 104)
Format of therapy and exercises			
Paper copy plus CD	−0.005	0.965	−7(−308 to 295)
Paper copy plus DVD	0.061	0.616	78 (−227 to 384)
Paper copy plus website	0.110	0.375	141 (−172 to 455)
Paper copy plus smartphone application	0.168	0.182	216 (−105 to 537)
Cost and programme start			
National Health Service cost	−0.001*	0.000	–
Time to start of therapy	−0.043	0.166	−56(−135 to 24)
Therapy provision			
Constant for Therapy A	1.950*	0.000	2515 (1843 to 3188)
Constant for Therapy B	1.350*	0.000	1741 (1153 to 2328)

*Statistical significance (p<0.05)

The significant positive constants for Therapy A and Therapy B estimated in the conditional logistic regression suggest that participants highly valued receiving therapy (compared with no therapy). Significant results were indicated for two factors; participants disliked having no information about the therapy before it started and disliked therapy options which had a higher cost impact for the NHS. While results indicate preferences are stronger for home-based therapy options, few of the other coefficients in the model were statistically significant, indicating that there were not strong preferences within the pilot sample for therapy design elements. This was to be expected given the pilot design and sample size. A larger sample size is required to better estimate preferences for therapy design to ensure it is designed to best meet patients’ needs.

### Sample size estimation for a full study

To estimate the required sample size for a full study of patients’ preferences for a home-based psychological therapy, the coefficients estimated in the model in this pilot study were used as prior values to design optimal D-efficient and Bayesian experimental designs. Table 4 outlines the estimated minimum sample sizes to estimate each coefficient.

**Table 4:** Sample size requirements

Attribute/level	Expected sample size required to estimate parameter by design type	
	D-Efficient	Bayesian
Psychological intervention		
Home-based with telephone support	290	308
Home-based with face-to-face support	245	251
Group face-to-face in primary or community care	370	362
Group face-to-face in secondary care	227	228
Information provided		
No information	41	41
Printed leaflet	126 596	120 853
Overview from a healthcare professional	304	321
Overview from a healthcare professional plus printed leaflet	89	87
Format of the therapy manual and exercises		
Paper copy plus CD	39 768	39 841
Paper copy plus DVD	960	924
Paper copy plus website	179	190
Paper copy plus smartphone application	104	100
Additional cost to the National Health Service	2	2
Programme start	25	24

It is unlikely that the coefficients for printed leaflet or information in a paper copy with a CD could be estimated with a feasible sample size, suggesting it may be worth excluding or redesigning this in future research. However, most coefficients could be estimated with a sample size of 370 or 362 depending on the whether a D-efficient design or Bayesian design was used.

### Participant comments

The mean time to complete the survey reported by participants was 23.87 min (SD 14.20; range 5–80).

Fifteen participants left comments on the questionnaire, these were often just general thoughts around CR. The most common comment specific to the DCE was around the cost attribute, and how participants considered cost to be very important, for example,

The treatments are far too costly!

In each answer I've taken into account the cost to the NHS. Any form of affordable help by the NHS is preferable to no help at all.

One participant commented that they volunteer at a CR centre and related it to how much people value talking to people with lived experience (peer support). While not captured in this questionnaire (peer group support), it is an option within a separate DCE being undertaken by our research team related to the clinic-based group-MCT trial.^17 18^ This information could also prompt ideas for future research, for example, how could peer support be effectively included in home-based CR interventions.

One comment highlighted that in the chosen sample we might have an issue of adaptation.

As I now feel well. I walk every day even in lock down. I don’t feel I need any psycholical(sic) help. It was 3 years ago when I had the heart attack. At the time I felt anxious and I could have benefited from help at the time.

People adapt to their illnesses, especially chronic conditions, and this adaptation may affect how they value their health and choices they make about healthcare.^35 36^ This participant describes how they feel psychological support is no longer relevant to them (and subsequently they used the opt-out), but that when the problem first occurred, they would have felt differently; this is likely to mean that their preferences have changed.

Three participants commented on the repetitiveness of the choice sets. An alternative to consider and assess in future research is a blocked design with fewer questions per participant. A single participant noted that there was a lot of information to read up front and that reducing this would be useful, however, this is partly dictated by ethics requirements.

## Discussion

The study aimed to evaluate the feasibility of conducting a DCE to identify preferences for psychological therapy delivery for people who were eligible to attend CR with symptoms of depression and/or anxiety, and to provide an initial indication of preferences. It demonstrated that conducting a survey of this type is feasible, and the study provides an estimate of the sample size needed for a full (larger) study. The initial findings, limited by sample size, indicate that participants tend to prefer home-based therapy, however this was not statistically significant. Two statistically significant findings were identified; participants were keen to receive some information about psychological treatment prior to therapy and preferred lower costs. An opt-out was included but the coefficients indicated that participants would prefer to partake in a therapy versus no therapy.

The participants did not want to receive no information about psychological therapy prior to starting therapy (statistically significant), which highlights a need to provide people with enough information to allow them to make informed choices. The strongest, but not statistically significant, preferences were for receiving an overview of therapy from a healthcare professional. There was some evidence to suggest preferences were stronger for having a paper copy of the manual plus a smartphone application. A published study found technology delivered intervention would be less popular, however, this focused on exercise and educational sessions.^10^ A questionnaire conducted in people attending CR found respondents were interested in CR delivered via the internet or mobile phones, although interest decreased with age.^37^ Preferences indicted people would prefer therapy to start sooner after hospital discharge, which aligns with a previous study (not specific to psychological therapy) which found stronger preferences for CR starting within 2 weeks.^10^ Participants disliked options with a higher cost to the NHS (statistically significant) which was also reflected in their comments. This aligns with the wider literature, as cost attributes were noted to be important in a systematic review of preference research in anxiety and depression.^38^

There was a reasonable response rate to the survey (39%) given that the first invitation was sent just before the COVID-19 pandemic resulted in a UK shutdown. The PPI group were consulted about the response rate and suggested that the target population were more likely to be shielding compared with the general population and subsequently would be hesitant to leave their home for non-essentials (eg, posting the survey back). It was suggested (by the PPI group) that the pandemic may have had varied effects on potential participants; for some people it could have made them more apathetic towards research due to the negative mental health effects, but for others it could have increased engagement as they looked for activities to avoid boredom. Participants were recruited from a trial sample for this pilot, and findings confirm a larger study is possible. Future studies do not need to restrict to trial recruitment and could consider other options (eg, online panels) which may make recruiting a larger sample easier.

There are several limitations to this research. Most substantially, it is limited by the sample size and the lack of diversity in the sample. The number and type of participants recruited for this study are likely to have affected the elicited preferences. As it is a pilot, the sample size is small and restricts the results. Moreover, the type of participants recruited, and the timing of recruitment may have affected preferences. It is possible that selection bias affected the results. We recruited from a trial sample of participants who had experienced symptoms of anxiety and/or depression when they attended CR, this sample may be more favourable towards receiving psychological therapy compared with the general CR population. Participants had symptoms of anxiety and/or depression when they were recruited to the trial, though as the DCE took place after the end of trial follow-up participants may have recovered from their symptoms. A minority (39%) of the pilot trial participants took part and we do not have any information of the remaining trial participants to explain why they did not return surveys. As mentioned, the study recruited during the COVID-19 pandemic which may have influenced the response rate and the responses, limiting generalisability. The PPI group noted that preferences may have been affected by local and national lockdown restrictions, which may strengthen preferences for home-based intervention. However, the PPI group also discussed how challenging it could be to make time and to share spaces with family members during the pandemic, in a way that would allow someone to partake in home-based psychological therapy privately. In relation to preferences for technology, the group had mixed opinions; with some members commenting on becoming more confident with technology and others experiencing technology fatigue during lockdown.

Many of the previous studies noted that preference heterogeneity affected their results, including whether participants preferred home-based interventions and preferences for technology.^10 12–14^ Unfortunately, this could not be investigated given our sample size and may explain in part the lack of significant results. The participant group who responded to the survey were quite homogeneous (eg, majority white males aged over 55 and retired), which while fairly reflective of the population accessing CR affects the ability to investigate which patient characteristics are tied to preferences. The National Audit of CR discussed that while over 30% of patients registered are over 75 and patients have a mean age of 67 (range 18–105), RCTs tend to consider younger age groups.^4^ This issue is present in our sample, as only a single participant was aged over 75. Further preference research could benefit from increasing recruitment in older age categories. The survey had a higher proportion of female responses compared with patients accessing CR (27.8% females in England) but reflected the population in the feasibility trial.^4^ One of the comments received from a participant prompted us to consider whether this sample was the most relevant for the study. We invited trial participants and while these are a very relevant group, as they have lived experience, they are less heterogeneous compared with the population who will be offered CR. Furthermore, as highlighted by the participant comment, our participants experienced their cardiac event years previously, and may have recovered or adapted to their condition which could affect their preferences. Further research could explore this by using a larger sample that included other relevant groups (eg, the general public or people who have very recently experienced a cardiac event).

Future research should be wary of the length of survey materials. Participants were asked to respond to 16 choice set questions and some comments noted that the survey felt repetitive. In future, a blocking design may help to reduce the burden of completion and could also help to reduce the sample size needed. Furthermore, we would encourage future researchers to recruit a larger and more varied sample of participants. In particular, we would encourage research to explore whether the timing of preference research affects preferences, for example, whether people have yet to attend or have completed CR. Exploring the association between the severity of depression and/or anxiety, and previous history of depression and/or anxiety, would help to identify preference heterogeneity between groups with and without experience of mental illness. DCEs are limited in terms of the number of attributes and levels that can feasibly be included; a review found a median of five attributes included.^8^ This pilot study included attributes based on a qualitative feedback and PPI feedback, as well as discussion with the trial research team. Given the heterogeneity in the delivery of CR and the range of psychological interventions that could be implemented, future research could add or use attributes focusing on more specific details of psychological therapy.^6 39^ Preference research should be considered alongside evidence on the clinical and cost-effectiveness of additions to CR.

While there are a number of limitations, the pilot study fulfils its objectives by establishing the feasibility of a DCE in this participant group; identifying potential attributes, levels and sample sizes and patient groups. As well as providing some information on the potential strengths of preferences for different attributes and levels. To the authors’ knowledge this is the first DCE to examine the preferred delivery of psychological therapy in the population attending CR. It demonstrates the potential of research of this type in informing future intervention design, however more research, in particular larger studies with more diverse samples, is needed.

## Conclusions

This pilot study demonstrates the feasibility of conducting DCEs in this participant group and provides initial evidence for preferences related to the delivery of psychological therapy in CR. Statistically significant results demonstrated participants wanted to receive some information about psychological therapy prior to starting therapy and preferred to receive options with a lower cost to the NHS. Furthermore, the sampled participants tend to favour home-based psychological therapy in CR, with quicker start times and online or smartphone assisted therapy, though these results were not statistically significant. Finally, results indicated that participants would prefer to partake in a therapy compared with no therapy. Results are limited due to the sample recruited. Further research is needed to strengthen conclusions and investigate preference heterogeneity.

## Data Availability

Anonymised data are available on reasonable request from the corresponding author.
