# Supplementary material for: Delivery preferences for psychological intervention in cardiac rehabilitation: a pilot discrete choice experiment
Source: Open Heart. 2021 Aug 23;8(2):e001747. doi: 10.1136/openhrt-2021-001747 (PMC8383873; doi:10.1136/openhrt-2021-001747)
Supplement: Supplementary data [file openhrt-2021-001747supp001.pdf]

Correspondence to: gemma.shields@manchester.ac.uk

Delivery Preferences for Psychological Intervention in Cardiac Rehabilitation: A Pilot Discrete Choice Experiment

Example questionnaire

The MCT Pathway project is funded by the National Institute for Health Research (NIHR) under its Programme Grants for Applied Research Programme (Reference Number RP-PG-1211-20011).

Survey

Section A – General information

First we would like to ask some questions about you. These details will be kept confidential if you choose to answer. Please tick or add text to the appropriate box to indicate your response.

A1. How old are you?

- 18-24 ☐
- 25-34 ☐
- 35-44 ☐
- 45-54 ☐
- 55-64 ☐
- 65-74 ☐
- 75-84 ☐
- 85+ ☐
- Prefer not to answer ☐

A2. How would you describe your gender?

- Male ☐
- Female ☐
- In another way ☐
- Prefer not to answer ☐

A3. What is your ethnic group?

- White ☐
- Mixed/multiple ethnic groups ☐
- Asian/Asian British ☐
- Black/African/Caribbean/Black British ☐
- Other ethnic group ☐

A4. Which of the following categories best describes your employment

- Paid employment (including part time or full time) ☐
- Unpaid employment/activities (including voluntary employment, study, housewife/husband, retired) ☐
- Unemployed ☐
- Prefer not to answer ☐

A5. What is your education level?

- GCSE (or equivalent) or above ☐
- No GCSE (or equivalent) ☐
- Prefer not to answer ☐

Correspondence to: gemma.shields@manchester.ac.uk

The MCT Pathway project is funded by the National Institute for Health Research (NIHR) under its Programme Grants for Applied Research Programme (Reference Number RP-PG-1211-20011).

A6. Are you a healthcare professional working in cardiac rehabilitation?

Yes ☐
No ☐

A7. Have you been affected by cardiac problems?

Yes (have undertaken cardiac rehabilitation) ☐
Yes (have been offered cardiac rehabilitation, but not taken it) ☐
Yes (have not been offered cardiac rehabilitation) ☐
No ☐
Prefer not to answer ☐

If you answered yes to question A7, please tell use more about the cardiac problem below, if not skip to question A10.

A8. What is/was the cardiac problem/s?

Please describe the cardiac problem/s ☐

A9. When did the problem/s first occur?

Record the years and months since the problem first occured: years months

A10. Has anyone in your family or close to you been affected by cardiac problems?

Yes ☐
No ☐
Don't know ☐
Prefer not to answer ☐

A11. Do you have prior experience of psychological therapy?

Yes ☐
No ☐
Prefer not to answer ☐

If you answered yes to question A11, please tell use more about your experience of psychological therapy below, if not skip to section B.

A12. Which types of psychological therapies have you experienced?

Group psychological therapy ☐
One-to-one psychological therapy ☐

Correspondence to: gemma.shields@manchester.ac.uk

The MCT Pathway project is funded by the National Institute for Health Research (NIHR) under its Programme Grants for Applied Research Programme (Reference Number RP-PG-1211-20011).

Section B – Your Health Today

In this section, we ask about your overall health today. Under each heading, please tick the **one** box that best describes your health **today.**

B1. Mobility

- I have no problems in walking about ☐
- I have slight problems in walking about ☐
- I have moderate problems in walking about ☐
- I have severe problems in walking about ☐
- I am unable to walk about ☐

B2. Self-Care

- I have no problems washing or dressing myself ☐
- I have slight problems washing or dressing myself ☐
- I have moderate problems washing or dressing myself ☐
- I have severe problems washing or dressing myself ☐
- I am unable to wash or dress myself ☐

B3. Usual Activities (e.g. *work, housework, family or leisure activities*)

- I have no problems doing my usual activities ☐
- I have slight problems doing my usual activities ☐
- I have moderate problems doing my usual activities ☐
- I have severe problems doing my usual activities ☐
- I am unable to do my usual activities ☐

B4. Pain/Discomfort

- I have no pain or discomfort ☐
- I have slight pain or discomfort ☐
- I have moderate pain or discomfort ☐
- I have severe pain or discomfort ☐
- I have extreme pain or discomfort ☐

B5. Anxiety/Depression

- I am not anxious or depressed ☐
- I am slightly anxious or depressed ☐
- I am moderately anxious or depressed ☐
- I am severely anxious or depressed ☐
- I am extremely anxious or depressed ☐

Correspondence to: gemma.shields@manchester.ac.uk

The MCT Pathway project is funded by the National Institute for Health Research (NIHR) under its Programme Grants for Applied Research Programme (Reference Number RP-PG-1211-20011).

Section C – Hypothetical Psychological Therapy Alternatives

The next section of questions ask you to compare possible descriptions of different psychological therapies and to choose which you prefer by ticking a box to indicate your choice. Following your choice you can indicate whether you would take part in your choice or whether you would actually opt out of partaking in psychological therapy. There are 16 of these questions. There are no right or wrong answers. But if you are unsure or have problems answering these questions, please do feel free to contact the research team for help with the questionnaire. Contact details are provided on the instructions. Please try to answer all questions.

Correspondence to: gemma.shields@manchester.ac.uk

The MCT Pathway project is funded by the National Institute for Health Research (NIHR) under its Programme Grants for Applied Research Programme (Reference Number RP-PG-1211-20011).

C1. Two potential psychological therapies are described below. Remember these would be received in addition to the standard cardiac rehabilitation package. The statements on the left describe different delivery of the therapy. The statements on the right describe the different options. Imagine that you are offered the choice between therapy A and B. Taking everything into account which therapy would you prefer? Choose which therapy you prefer by ticking the box under therapy A and therapy B. There are no right or wrong answers, it is your view that is important.

Delivery	Therapy A	Therapy B
Psychological intervention to be received alongside your standard cardiac rehabilitation programme	Home-based psychological therapy using a manual with occasional telephone support from a healthcare professional	Home-based psychological therapy using a manual with occasional face to face support from a healthcare professional
The information given to you prior to accepting and starting treatment that gives you an idea of what to expect from the therapy	No information provided	A printed leaflet of information
Format of the therapy manual and exercises	Printed (paper copy) of the therapy manual and an accompanying audio CD of exercises	Printed (paper copy) of the therapy manual and an accompanying DVD (video) of exercises
Additional cost to the NHS	£0	£500
Programme start	Within 2 weeks of hospital discharge	Within 4 weeks of hospital discharge
**C1.1. Of the options presented above which do you like most** *(Tick one)*	☐	☐

**C1.2. If you had to choose from the option that you like the most, or no psychological therapy included in your cardiac rehabilitation pathway, which would you choose?** *(Tick one)*	
The option I chose above	☐
No psychological therapy	☐

Correspondence to: gemma.shields@manchester.ac.uk

The MCT Pathway project is funded by the National Institute for Health Research (NIHR) under its Programme Grants for Applied Research Programme (Reference Number RP-PG-1211-20011).

C2. Two potential psychological therapies are described below. Remember these would be received in addition to the standard cardiac rehabilitation package. The statements on the left describe different delivery of the therapy. The statements on the right describe the different options. Imagine that you are offered the choice between therapy A and B. Taking everything into account which therapy would you prefer? Choose which therapy you prefer by ticking the box under therapy A and therapy B. There are no right or wrong answers, it is your view that is important.

**Delivery**	**Therapy A**	**Therapy B**
Psychological intervention to be received alongside your standard cardiac rehabilitation programme	Home-based psychological therapy using a manual with occasional telephone support from a healthcare professional	Home-based psychological therapy using a manual with occasional face to face support from a healthcare professional
The information given to you prior to accepting and starting treatment that gives you an idea of what to expect from the therapy	A printed leaflet of information	An overview of the therapy from a healthcare provider with a chance to ask questions
Format of the therapy manual and exercises	Printed (paper copy) of the therapy manual and an accompanying DVD (video) of exercises	Printed (paper copy) plus a website based manual and exercises
Additional cost to the NHS	£500	£1000
Programme start	Within 4 weeks of hospital discharge	Within 6 weeks of hospital discharge
**C2.1. Of the options presented above which do you like most** *(Tick one)*	☐	☐

**C2.2. If you had to choose from the option that you like the most, or no psychological therapy included in your cardiac rehabilitation pathway, which would you choose?** *(Tick one)*	
The option I chose above	☐
No psychological therapy	☐

Correspondence to: gemma.shields@manchester.ac.uk

The MCT Pathway project is funded by the National Institute for Health Research (NIHR) under its Programme Grants for Applied Research Programme (Reference Number RP-PG-1211-20011).

C3. Two potential psychological therapies are described below. Remember these would be received in addition to the standard cardiac rehabilitation package. The statements on the left describe different delivery of the therapy. The statements on the right describe the different options. Imagine that you are offered the choice between therapy A and B. Taking everything into account which therapy would you prefer? Choose which therapy you prefer by ticking the box under therapy A and therapy B. There are no right or wrong answers, it is your view that is important.

**Delivery**	**Therapy A**	**Therapy B**
Psychological intervention to be received alongside your standard cardiac rehabilitation programme	Home-based psychological therapy using a manual with occasional telephone support from a healthcare professional	Home-based psychological therapy using a manual with occasional face to face support from a healthcare professional
The information given to you prior to accepting and starting treatment that gives you an idea of what to expect from the therapy	An overview of the therapy from a healthcare provider with a chance to ask questions	An overview of the therapy from a healthcare provider with a chance to ask questions and a printed leaflet
Format of the therapy manual and exercises	Printed (paper copy) plus a website based manual and exercises	Printed (paper copy) plus a smartphone application-based manual and exercises
Additional cost to the NHS	£1000	£2000
Programme start	Within 6 weeks of hospital discharge	Within 8 weeks of hospital discharge
**C3.1. Of the options presented above which do you like most** *(Tick one)*	☐	☐

**C3.2. If you had to choose from the option that you like the most, or no psychological therapy included in your cardiac rehabilitation pathway, which would you choose?** *(Tick one)*	
The option I chose above	☐
No psychological therapy	☐

Correspondence to: gemma.shields@manchester.ac.uk

The MCT Pathway project is funded by the National Institute for Health Research (NIHR) under its Programme Grants for Applied Research Programme (Reference Number RP-PG-1211-20011).

C4. Two potential psychological therapies are described below. Remember these would be received in addition to the standard cardiac rehabilitation package. The statements on the left describe different delivery of the therapy. The statements on the right describe the different options. Imagine that you are offered the choice between therapy A and B. Taking everything into account which therapy would you prefer? Choose which therapy you prefer by ticking the box under therapy A and therapy B. There are no right or wrong answers, it is your view that is important.

**Delivery**	**Therapy A**	**Therapy B**
Psychological intervention to be received alongside your standard cardiac rehabilitation programme	Home-based psychological therapy using a manual with occasional telephone support from a healthcare professional	Home-based psychological therapy using a manual with occasional face to face support from a healthcare professional
The information given to you prior to accepting and starting treatment that gives you an idea of what to expect from the therapy	An overview of the therapy from a healthcare provider with a chance to ask questions and a printed leaflet	No information provided
Format of the therapy manual and exercises	Printed (paper copy) plus a smartphone application-based manual and exercises	Printed (paper copy) of the therapy manual and an accompanying audio CD of exercises
Additional cost to the NHS	£2000	£0
Programme start	Within 8 weeks of hospital discharge	Within 2 weeks of hospital discharge
**C4.1. Of the options presented above which do you like most** *(Tick one)*	☐	☐

**C4.2. If you had to choose from the option that you like the most, or no psychological therapy included in your cardiac rehabilitation pathway, which would you choose?** *(Tick one)*	
The option I chose above	☐
No psychological therapy	☐

Correspondence to: gemma.shields@manchester.ac.uk

The MCT Pathway project is funded by the National Institute for Health Research (NIHR) under its Programme Grants for Applied Research Programme (Reference Number RP-PG-1211-20011).

C5. Two potential psychological therapies are described below. Remember these would be received in addition to the standard cardiac rehabilitation package. The statements on the left describe different delivery of the therapy. The statements on the right describe the different options. Imagine that you are offered the choice between therapy A and B. Taking everything into account which therapy would you prefer? Choose which therapy you prefer by ticking the box under therapy A and therapy B. There are no right or wrong answers, it is your view that is important.

**Delivery**	**Therapy A**	**Therapy B**
Psychological intervention to be received alongside your standard cardiac rehabilitation programme	Home-based psychological therapy using a manual with occasional face to face support from a healthcare professional	Group psychological therapy based in primary or community care (e.g. a local GP or NHS clinic) delivered by a healthcare professional
The information given to you prior to accepting and starting treatment that gives you an idea of what to expect from the therapy	No information provided	A printed leaflet of information
Format of the therapy manual and exercises	Printed (paper copy) of the therapy manual and an accompanying DVD (video) of exercises	Printed (paper copy) plus a website based manual and exercises
Additional cost to the NHS	£1000	£2000
Programme start	Within 8 weeks of hospital discharge	Within 2 weeks of hospital discharge
**C5.1. Of the options presented above which do you like most** *(Tick one)*	☐	☐

**C5.2. If you had to choose from the option that you like the most, or no psychological therapy included in your cardiac rehabilitation pathway, which would you choose?** *(Tick one)*	
The option I chose above	☐
No psychological therapy	☐

Correspondence to: gemma.shields@manchester.ac.uk

The MCT Pathway project is funded by the National Institute for Health Research (NIHR) under its Programme Grants for Applied Research Programme (Reference Number RP-PG-1211-20011).

C6. Two potential psychological therapies are described below. Remember these would be received in addition to the standard cardiac rehabilitation package. The statements on the left describe different delivery of the therapy. The statements on the right describe the different options. Imagine that you are offered the choice between therapy A and B. Taking everything into account which therapy would you prefer? Choose which therapy you prefer by ticking the box under therapy A and therapy B. There are no right or wrong answers, it is your view that is important.

**Delivery**	**Therapy A**	**Therapy B**
Psychological intervention to be received alongside your standard cardiac rehabilitation programme	Home-based psychological therapy using a manual with occasional face to face support from a healthcare professional	Group psychological therapy based in primary or community care (e.g. a local GP or NHS clinic) delivered by a healthcare professional
The information given to you prior to accepting and starting treatment that gives you an idea of what to expect from the therapy	A printed leaflet of information	An overview of the therapy from a healthcare provider with a chance to ask questions
Format of the therapy manual and exercises	Printed (paper copy) of the therapy manual and an accompanying audio CD of exercises	Printed (paper copy) of the therapy manual and an accompanying DVD (video) of exercises
Additional cost to the NHS	£2000	£0
Programme start	Within 6 weeks of hospital discharge	Within 8 weeks of hospital discharge
**C6.1. Of the options presented above which do you like most** *(Tick one)*	☐	☐

**C6.2. If you had to choose from the option that you like the most, or no psychological therapy included in your cardiac rehabilitation pathway, which would you choose?** *(Tick one)*	
The option I chose above	☐
No psychological therapy	☐

Correspondence to: gemma.shields@manchester.ac.uk

The MCT Pathway project is funded by the National Institute for Health Research (NIHR) under its Programme Grants for Applied Research Programme (Reference Number RP-PG-1211-20011).

C7. Two potential psychological therapies are described below. Remember these would be received in addition to the standard cardiac rehabilitation package. The statements on the left describe different delivery of the therapy. The statements on the right describe the different options. Imagine that you are offered the choice between therapy A and B. Taking everything into account which therapy would you prefer? Choose which therapy you prefer by ticking the box under therapy A and therapy B. There are no right or wrong answers, it is your view that is important.

Delivery	Therapy A	Therapy B
Psychological intervention to be received alongside your standard cardiac rehabilitation programme	Home-based psychological therapy using a manual with occasional face to face support from a healthcare professional	Group psychological therapy based in primary or community care (e.g. a local GP or NHS clinic) delivered by a healthcare professional
The information given to you prior to accepting and starting treatment that gives you an idea of what to expect from the therapy	An overview of the therapy from a healthcare provider with a chance to ask questions	An overview of the therapy from a healthcare provider with a chance to ask questions and a printed leaflet
Format of the therapy manual and exercises	Printed (paper copy) plus a smartphone application-based manual and exercises	Printed (paper copy) of the therapy manual and an accompanying audio CD of exercises
Additional cost to the NHS	£0	£500
Programme start	Within 4 weeks of hospital discharge	Within 6 weeks of hospital discharge
**C7.1. Of the options presented above which do you like most** *(Tick one)*	☐	☐

**C7.2. If you had to choose from the option that you like the most, or no psychological therapy included in your cardiac rehabilitation pathway, which would you choose?** *(Tick one)*	
The option I chose above	☐
No psychological therapy	☐

Correspondence to: gemma.shields@manchester.ac.uk

The MCT Pathway project is funded by the National Institute for Health Research (NIHR) under its Programme Grants for Applied Research Programme (Reference Number RP-PG-1211-20011).

C8. Two potential psychological therapies are described below. Remember these would be received in addition to the standard cardiac rehabilitation package. The statements on the left describe different delivery of the therapy. The statements on the right describe the different options. Imagine that you are offered the choice between therapy A and B. Taking everything into account which therapy would you prefer? Choose which therapy you prefer by ticking the box under therapy A and therapy B. There are no right or wrong answers, it is your view that is important.

Delivery	Therapy A	Therapy B
Psychological intervention to be received alongside your standard cardiac rehabilitation programme	Home-based psychological therapy using a manual with occasional face to face support from a healthcare professional	Group psychological therapy based in primary or community care (e.g. a local GP or NHS clinic) delivered by a healthcare professional
The information given to you prior to accepting and starting treatment that gives you an idea of what to expect from the therapy	An overview of the therapy from a healthcare provider with a chance to ask questions and a printed leaflet	No information provided
Format of the therapy manual and exercises	Printed (paper copy) plus a website based manual and exercises	Printed (paper copy) plus a smartphone application-based manual and exercises
Additional cost to the NHS	£500	£1000
Programme start	Within 2 weeks of hospital discharge	Within 4 weeks of hospital discharge
**C8.1. Of the options presented above which do you like most** *(Tick one)*	☐	☐

**C8.2. If you had to choose from the option that you like the most, or no psychological therapy included in your cardiac rehabilitation pathway, which would you choose?** *(Tick one)*	
The option I chose above	☐
No psychological therapy	☐

Correspondence to: gemma.shields@manchester.ac.uk

The MCT Pathway project is funded by the National Institute for Health Research (NIHR) under its Programme Grants for Applied Research Programme (Reference Number RP-PG-1211-20011).

C9. Two potential psychological therapies are described below. Remember these would be received in addition to the standard cardiac rehabilitation package. The statements on the left describe different delivery of the therapy. The statements on the right describe the different options. Imagine that you are offered the choice between therapy A and B. Taking everything into account which therapy would you prefer? Choose which therapy you prefer by ticking the box under therapy A and therapy B. There are no right or wrong answers, it is your view that is important.

Delivery	Therapy A	Therapy B
Psychological intervention to be received alongside your standard cardiac rehabilitation programme	Group psychological therapy based in primary or community care (e.g. a local GP or NHS clinic) delivered by a healthcare professional	Group psychological therapy based in secondary care (e.g. at a hospital) delivered by a healthcare professional
The information given to you prior to accepting and starting treatment that gives you an idea of what to expect from the therapy	No information provided	A printed leaflet of information
Format of the therapy manual and exercises	Printed (paper copy) plus a website based manual and exercises	Printed (paper copy) plus a smartphone application-based manual and exercises
Additional cost to the NHS	£2000	£0
Programme start	Within 4 weeks of hospital discharge	Within 6 weeks of hospital discharge
**C9.1. Of the options presented above which do you like most** *(Tick one)*	☐	☐

**C9.2. If you had to choose from the option that you like the most, or no psychological therapy included in your cardiac rehabilitation pathway, which would you choose?** *(Tick one)*	
The option I chose above	☐
No psychological therapy	☐

Correspondence to: gemma.shields@manchester.ac.uk

The MCT Pathway project is funded by the National Institute for Health Research (NIHR) under its Programme Grants for Applied Research Programme (Reference Number RP-PG-1211-20011).

C10. Two potential psychological therapies are described below. Remember these would be received in addition to the standard cardiac rehabilitation package. The statements on the left describe different delivery of the therapy. The statements on the right describe the different options. Imagine that you are offered the choice between therapy A and B. Taking everything into account which therapy would you prefer? Choose which therapy you prefer by ticking the box under therapy A and therapy B. There are no right or wrong answers, it is your view that is important.

**Delivery**	**Therapy A**	**Therapy B**
Psychological intervention to be received alongside your standard cardiac rehabilitation programme	Group psychological therapy based in primary or community care (e.g. a local GP or NHS clinic) delivered by a healthcare professional	Group psychological therapy based in secondary care (e.g. at a hospital) delivered by a healthcare professional
The information given to you prior to accepting and starting treatment that gives you an idea of what to expect from the therapy	A printed leaflet of information	An overview of the therapy from a healthcare provider with a chance to ask questions
Format of the therapy manual and exercises	Printed (paper copy) plus a smartphone application-based manual and exercises	Printed (paper copy) of the therapy manual and an accompanying audio CD of exercises
Additional cost to the NHS	£1000	£2000
Programme start	Within 2 weeks of hospital discharge	Within 4 weeks of hospital discharge
**C10.1. Of the options presented above which do you like most** *(Tick one)*	☐	☐

**C10.2. If you had to choose from the option that you like the most, or no psychological therapy included in your cardiac rehabilitation pathway, which would you choose?** *(Tick one)*	
The option I chose above	☐
No psychological therapy	☐

Correspondence to: gemma.shields@manchester.ac.uk

The MCT Pathway project is funded by the National Institute for Health Research (NIHR) under its Programme Grants for Applied Research Programme (Reference Number RP-PG-1211-20011).

C11. Two potential psychological therapies are described below. Remember these would be received in addition to the standard cardiac rehabilitation package. The statements on the left describe different delivery of the therapy. The statements on the right describe the different options. Imagine that you are offered the choice between therapy A and B. Taking everything into account which therapy would you prefer? Choose which therapy you prefer by ticking the box under therapy A and therapy B. There are no right or wrong answers, it is your view that is important.

**Delivery**	**Therapy A**	**Therapy B**
Psychological intervention to be received alongside your standard cardiac rehabilitation programme	Group psychological therapy based in primary or community care (e.g. a local GP or NHS clinic) delivered by a healthcare professional	Group psychological therapy based in secondary care (e.g. at a hospital) delivered by a healthcare professional
The information given to you prior to accepting and starting treatment that gives you an idea of what to expect from the therapy	An overview of the therapy from a healthcare provider with a chance to ask questions	An overview of the therapy from a healthcare provider with a chance to ask questions and a printed leaflet
Format of the therapy manual and exercises	Printed (paper copy) of the therapy manual and an accompanying audio CD of exercises	Printed (paper copy) of the therapy manual and an accompanying DVD (video) of exercises
Additional cost to the NHS	£500	£1000
Programme start	Within 8 weeks of hospital discharge	Within 2 weeks of hospital discharge
**C11.1. Of the options presented above which do you like most** *(Tick one)*	☐	☐

**C11.2. If you had to choose from the option that you like the most, or no psychological therapy included in your cardiac rehabilitation pathway, which would you choose?** *(Tick one)*	
The option I chose above	☐
No psychological therapy	☐

Correspondence to: gemma.shields@manchester.ac.uk

The MCT Pathway project is funded by the National Institute for Health Research (NIHR) under its Programme Grants for Applied Research Programme (Reference Number RP-PG-1211-20011).

C12. Two potential psychological therapies are described below. Remember these would be received in addition to the standard cardiac rehabilitation package. The statements on the left describe different delivery of the therapy. The statements on the right describe the different options. Imagine that you are offered the choice between therapy A and B. Taking everything into account which therapy would you prefer? Choose which therapy you prefer by ticking the box under therapy A and therapy B. There are no right or wrong answers, it is your view that is important.

Delivery	Therapy A	Therapy B
Psychological intervention to be received alongside your standard cardiac rehabilitation programme	Group psychological therapy based in primary or community care (e.g. a local GP or NHS clinic) delivered by a healthcare professional	Group psychological therapy based in secondary care (e.g. at a hospital) delivered by a healthcare professional
The information given to you prior to accepting and starting treatment that gives you an idea of what to expect from the therapy	An overview of the therapy from a healthcare provider with a chance to ask questions and a printed leaflet	No information provided
Format of the therapy manual and exercises	Printed (paper copy) of the therapy manual and an accompanying DVD (video) of exercises	Printed (paper copy) plus a website based manual and exercises
Additional cost to the NHS	£0	£500
Programme start	Within 6 weeks of hospital discharge	Within 8 weeks of hospital discharge
**C12.1. Of the options presented above which do you like most** *(Tick one)*	☐	☐

**C12.2. If you had to choose from the option that you like the most, or no psychological therapy included in your cardiac rehabilitation pathway, which would you choose?** *(Tick one)*	
The option I chose above	☐
No psychological therapy	☐

Correspondence to: gemma.shields@manchester.ac.uk

The MCT Pathway project is funded by the National Institute for Health Research (NIHR) under its Programme Grants for Applied Research Programme (Reference Number RP-PG-1211-20011).

C13. Two potential psychological therapies are described below. Remember these would be received in addition to the standard cardiac rehabilitation package. The statements on the left describe different delivery of the therapy. The statements on the right describe the different options. Imagine that you are offered the choice between therapy A and B. Taking everything into account which therapy would you prefer? Choose which therapy you prefer by ticking the box under therapy A and therapy B. There are no right or wrong answers, it is your view that is important.

Delivery	Therapy A	Therapy B
Psychological intervention to be received alongside your standard cardiac rehabilitation programme	Group psychological therapy based in secondary care (e.g. at a hospital) delivered by a healthcare professional	Home-based psychological therapy using a manual with occasional telephone support from a healthcare professional
The information given to you prior to accepting and starting treatment that gives you an idea of what to expect from the therapy	No information provided	A printed leaflet of information
Format of the therapy manual and exercises	Printed (paper copy) plus a smartphone application-based manual and exercises	Printed (paper copy) of the therapy manual and an accompanying audio CD of exercises
Additional cost to the NHS	£500	£1000
Programme start	Within 6 weeks of hospital discharge	Within 8 weeks of hospital discharge
**C13.1. Of the options presented above which do you like most** *(Tick one)*	☐	☐

**C13.2. If you had to choose from the option that you like the most, or no psychological therapy included in your cardiac rehabilitation pathway, which would you choose?** *(Tick one)*	
The option I chose above	☐
No psychological therapy	☐

Correspondence to: gemma.shields@manchester.ac.uk

The MCT Pathway project is funded by the National Institute for Health Research (NIHR) under its Programme Grants for Applied Research Programme (Reference Number RP-PG-1211-20011).

C14. Two potential psychological therapies are described below. Remember these would be received in addition to the standard cardiac rehabilitation package. The statements on the left describe different delivery of the therapy. The statements on the right describe the different options. Imagine that you are offered the choice between therapy A and B. Taking everything into account which therapy would you prefer? Choose which therapy you prefer by ticking the box under therapy A and therapy B. There are no right or wrong answers, it is your view that is important.

Delivery	Therapy A	Therapy B
Psychological intervention to be received alongside your standard cardiac rehabilitation programme	Group psychological therapy based in secondary care (e.g. at a hospital) delivered by a healthcare professional	Home-based psychological therapy using a manual with occasional telephone support from a healthcare professional
The information given to you prior to accepting and starting treatment that gives you an idea of what to expect from the therapy	A printed leaflet of information	An overview of the therapy from a healthcare provider with a chance to ask questions
Format of the therapy manual and exercises	Printed (paper copy) plus a website based manual and exercises	Printed (paper copy) plus a smartphone application-based manual and exercises
Additional cost to the NHS	£0	£500
Programme start	Within 8 weeks of hospital discharge	Within 2 weeks of hospital discharge
**C14.1. Of the options presented above which do you like most** *(Tick one)*	☐	☐

**C14.2. If you had to choose from the option that you like the most, or no psychological therapy included in your cardiac rehabilitation pathway, which would you choose?** *(Tick one)*	
The option I chose above	☐
No psychological therapy	☐

Correspondence to: gemma.shields@manchester.ac.uk

The MCT Pathway project is funded by the National Institute for Health Research (NIHR) under its Programme Grants for Applied Research Programme (Reference Number RP-PG-1211-20011).

C15. Two potential psychological therapies are described below. Remember these would be received in addition to the standard cardiac rehabilitation package. The statements on the left describe different delivery of the therapy. The statements on the right describe the different options. Imagine that you are offered the choice between therapy A and B. Taking everything into account which therapy would you prefer? Choose which therapy you prefer by ticking the box under therapy A and therapy B. There are no right or wrong answers, it is your view that is important.

Delivery	Therapy A	Therapy B
Psychological intervention to be received alongside your standard cardiac rehabilitation programme	Group psychological therapy based in secondary care (e.g. at a hospital) delivered by a healthcare professional	Home-based psychological therapy using a manual with occasional telephone support from a healthcare professional
The information given to you prior to accepting and starting treatment that gives you an idea of what to expect from the therapy	An overview of the therapy from a healthcare provider with a chance to ask questions	An overview of the therapy from a healthcare provider with a chance to ask questions and a printed leaflet
Format of the therapy manual and exercises	Printed (paper copy) of the therapy manual and an accompanying DVD (video) of exercises	Printed (paper copy) plus a website based manual and exercises
Additional cost to the NHS	£2000	£0
Programme start	Within 2 weeks of hospital discharge	Within 4 weeks of hospital discharge
**C15.1. Of the options presented above which do you like most** *(Tick one)*	☐	☐

**C15.2. If you had to choose from the option that you like the most, or no psychological therapy included in your cardiac rehabilitation pathway, which would you choose?** *(Tick one)*	
The option I chose above	☐
No psychological therapy	☐

Correspondence to: gemma.shields@manchester.ac.uk

The MCT Pathway project is funded by the National Institute for Health Research (NIHR) under its Programme Grants for Applied Research Programme (Reference Number RP-PG-1211-20011).

C16. Two potential psychological therapies are described below. Remember these would be received in addition to the standard cardiac rehabilitation package. The statements on the left describe different delivery of the therapy. The statements on the right describe the different options. Imagine that you are offered the choice between therapy A and B. Taking everything into account which therapy would you prefer? Choose which therapy you prefer by ticking the box under therapy A and therapy B. There are no right or wrong answers, it is your view that is important.

Delivery	Therapy A	Therapy B
Psychological intervention to be received alongside your standard cardiac rehabilitation programme	Group psychological therapy based in secondary care (e.g. at a hospital) delivered by a healthcare professional	Home-based psychological therapy using a manual with occasional telephone support from a healthcare professional
The information given to you prior to accepting and starting treatment that gives you an idea of what to expect from the therapy	An overview of the therapy from a healthcare provider with a chance to ask questions and a printed leaflet	No information provided
Format of the therapy manual and exercises	Printed (paper copy) of the therapy manual and an accompanying audio CD of exercises	Printed (paper copy) of the therapy manual and an accompanying DVD (video) of exercises
Additional cost to the NHS	£1000	£2000
Programme start	Within 4 weeks of hospital discharge	Within 6 weeks of hospital discharge
**C16.1. Of the options presented above which do you like most** *(Tick one)*	☐	☐

**C16.2. If you had to choose from the option that you like the most, or no psychological therapy included in your cardiac rehabilitation pathway, which would you choose?** *(Tick one)*	
The option I chose above	☐
No psychological therapy	☐

Correspondence to: gemma.shields@manchester.ac.uk

The MCT Pathway project is funded by the National Institute for Health Research (NIHR) under its Programme Grants for Applied Research Programme (Reference Number RP-PG-1211-20011).

Section D – Additional Information, Research and Feedback

Please provide us with any feedback that may help to improve future surveys.

D2. Further Comments

D1. How long did it take you to complete this survey (excluding breaks)?

Minutes ☐

If you have any comments about anything on this questionnaire, please write them here:

Correspondence to: gemma.shields@manchester.ac.uk

The MCT Pathway project is funded by the National Institute for Health Research (NIHR) under its Programme Grants for Applied Research Programme (Reference Number RP-PG-1211-20011).

Returning the completed questionnaire

Please return using the pre-paid envelope with the completed stated preference survey questionnaire to the MCT-Pathway study team at: PATHWAY Study, 3rd Floor Rawnsley Building, Manchester Royal Infirmary, Oxford Road, Manchester M13 9WL.

Alternatively, if you choose to complete the survey online, please follow the instructions to submit your answers.

Thank you for your help and taking the time to complete this questionnaire.

Correspondence to: gemma.shields@manchester.ac.uk

EQ-5D reporting across dimensions

Severity/ level	Mobility		Self-care		Usual activities		Pain/ discomfort		Anxiety/ depression	
	n	%	*n*	%	*n*	%	*n*	%	*n*	%
1	13	37.1	24	68.6	12	34.3	9	25.7	13	37.1
2	14	40.0	7	20.0	11	31.4	7	20.0	5	14.3
3	5	14.3	2	5.7	11	31.4	12	34.3	12	34.3
4	3	8.6	2	5.7	1	2.9	7	20.0	5	14.3
5	-	-	-	-	-	-	-	-	-	-
